# The epidemiology of bone cancer in 0 - 39 year olds in northern England, 1981 - 2002

**DOI:** 10.1186/1471-2407-10-357

**Published:** 2010-07-06

**Authors:** Rachel Eyre, Richard G Feltbower, Peter W James, Karen Blakey, Emmanuel Mubwandarikwa, David Forman, Patricia A McKinney, Mark S Pearce, Richard JQ McNally

**Affiliations:** 1 Institute of Health and Society, Newcastle University, Sir James Spence Institute, Royal Victoria Infirmary, Newcastle upon Tyne NE1 4LP, England, UK; 2 Paediatric Epidemiology Group, Centre for Epidemiology and Biostatistics, University of Leeds, Leeds LS2 9JT, England, UK; 3 Northern and Yorkshire Cancer Registry and Information Service, University of Leeds, Leeds LS9 7TF, England, UK; 4 Cancer Epidemiology Group, Leeds Institute of Genetics, Health & Therapeutics, Arthington House, Hospital Lane, Leeds LS16 6QB, England, UK; 5 Cancer Information Section, International Agency for Research on Cancer, 150, cours Albert Thomas, F-69372, Lyon, Cedex 08, France

## Abstract

**Background:**

There is a paucity of recent epidemiological data on bone cancers. The aim of this study was to describe incidence and survival patterns for bone cancers diagnosed during 1981 - 2002.

**Methods:**

Cases aged 0 - 39 years (236 osteosarcomas, 166 Ewing sarcomas and 73 chondrosarcomas) were analysed using Poisson and Cox regressions.

**Results:**

Incidence rates (per million persons per year) for osteosarcoma were 2.5 at age 0 - 14 years; 4.5 at age 15 - 29 years and 1.0 at age 30 - 39 years. Similarly, for Ewing sarcoma the incidence rates were 2.2; 2.9; 0.4 and for chondrosarcoma rates were 0.1; 1.2; 1.8 respectively. Incidence of osteosarcoma increased at an average annual rate of 2.5% (95% CI 0.4 - 4.7; *P *= 0.02), but there was no change in incidence of Ewing sarcoma or chondrosarcoma. There was a marginally statistically significant improvement in survival for Ewing sarcoma (hazard ratio (HR) per annum = 0.97; 95% CI 0.94 - 1.00; *P *= 0.06), although patients aged 15 - 39 years (n = 93) had worse overall survival than those aged 0 - 14 (n = 73; HR = 1.46; 95% CI 0.98 - 2.17; *P *= 0.06). There was no significant improvement in osteosarcoma survival (HR per annum = 0.98; 95% CI 0.95 - 1.01; *P *= 0.18).

**Conclusions:**

Reasons for poorer survival in Ewing sarcoma patients aged 15 - 39 years and failure to significantly improve survival for osteosarcoma patients requires further investigation.

## Background

Malignant bone tumours comprise 0.7% of all cancer diagnoses in the UK [1]. For all ages they have an age-standardised rate of 8 per 1,000,000 persons per year in males and 6 per 1,000,000 persons per year in females (standardised to the world population [2]). These tumours are the third most frequently occurring malignancy diagnosed in those aged 10 - 24 years and comprise more than twenty diagnostic sub-groups. Osteosarcoma is the most common sub-group, accounting for around a third of all diagnoses [3,4].

Primary bone cancer is rarely diagnosed in children before the age of five or adults over the age of sixty [3]. Incidence of osteosarcoma increases with age until a peak in late childhood or adolescence around the time of puberty, after which incidence declines. A second less pronounced peak occurs in older adults aged more than 65 years. Ewing sarcoma has an incidence peak in the late teenage years, whilst chondrosarcoma peaks in older adults (aged > 65 years) [4-6]. A small number of studies have explored the incidence and survival of childhood bone cancer [7-15]. However, even fewer studies have examined incidence and survival in those aged over 24 years.

We have previously analysed incidence and survival of bone cancer in children (aged 0 - 14 years) from northern England and the West Midlands [15]. The aim of the present study was to extend the age range analysed to include cases aged up to 39 years old. It is important to analyse this age-range as the incidence of both osteosarcoma and Ewing sarcoma peak after childhood (in those aged more than 15 years). We describe the incidence and survival from osteosarcoma, chondrosarcoma and Ewing sarcoma diagnosed in 0 - 39 year olds in northern England during the period 1981 - 2002 and compare patients aged 15 - 39 years with those aged 0 - 14 years. It should be noted that the childhood cases aged 0 - 14 years from the region were also included in the previous analysis (82 osteosarcoma; 73 Ewing sarcoma; 2 chondrosarcoma) [15]. The present study also updates previous analyses from northern England and the whole of Great Britain [9,16].

## Methods

### Study Subjects

Data were included for all patients aged 0 - 39 years registered during the period 1981 - 2002 by the Northern and Yorkshire Cancer Registry and Information Service (NYCRIS). This is a regional registry that records all cases of cancer and covers an area in the north of England with a population of approximately 6.6 million. The registry is population-based and has a very high level of ascertainment due to rigorous data collection procedures [17].

NYCRIS receives notifications of all malignancies diagnosed in the region from a variety of sources, the main source being pathology reports that are routinely submitted by all health care providers (National Health Service Trusts) in the area. Six months after diagnosis registry staff visit the hospital of primary treatment to manually extract additional clinical information from the patients' case notes. They check that tumour deaths correspond with the information on the original data source and amend when necessary.

In our study, records of primary malignant bone tumours registered between 1981 and 2002 in patients aged 0 - 39 years were extracted from NYCRIS. All tumours were categorised according to The World Health Organization Classification of Tumours system [18]. Secondary and benign bone tumours were excluded from this study. Information available for each case included demographic data (sex; date of birth); tumour diagnosis (date; site and morphology) and follow-up (current status; date of death; date of last follow-up). Analyses focussed on the three main diagnostic sub-groups: osteosarcoma; Ewing sarcoma and chondrosarcoma.

### Statistical Methods

Age-specific incidence rates per million persons per year were calculated based on annual mid-year population estimates for the study region obtained from Office for National Statistics. Comparisons of age-standardised incidence rates (ASRs) are only meaningful if they are standardised in the same way. The world standard has the most widespread use and calculation of world ASRs allows comparisons to be made with other published UK and international (especially European) studies [3,9,11,15]. ASRs were calculated using the age-specific incidence rates for five-year age groups weighted using the standard world population (originally proposed by Segi, but modified by Doll and colleagues and constructed from the pooled populations of forty six representative countries that had accurate population census data) [2,19-21]. Poisson regression was used to model the effects on incidence rate for time period of diagnosis (1981 - 1988, 1989 - 1995, 1996 - 2002); age group (0 - 14, 15 - 29, 30 - 39 years) and sex. Cases were followed up until 31^st ^December 2005. Five-year survival rates were calculated for all cases diagnosed during the period 1981 - 2000 as follow up was only available for all cases until the end of 2005. Survival rates were calculated using Kaplan-Meier estimation [22] and differences in survival between diagnostic groups (osteosarcoma, Ewing sarcoma and chondrosarcoma) assessed using log-rank tests. Cox Proportional Hazards regression analysis was used to model the probability of survival in relation to age, sex and year of diagnosis. Statistical significance was taken to be *P *< 0.05 and marginal significance as 0.05 ≤ *P *< 0.1 in all analyses. All statistical analyses were performed using Stata version 10.

## Results

There were 509 patients aged less than 40 years diagnosed with a malignant bone tumour in the NYCRIS area during the period 1981 to 2002. The number of cases and age-standardised incidence rates by diagnostic group, sex and age group are shown in Table 1. The most common bone cancer sub-groups diagnosed in 0 - 39 year olds were osteosarcoma (236 cases, by sub-type: 209 osteoblastic; 14 chondroblastic; 6 fibroblastic; 5 telangiectatic and 2 small cell) and Ewing sarcoma (166 cases). These comprised 46% and 33% of diagnoses respectively. Other sub-groups were diagnosed in smaller numbers with 73 chondrosarcoma cases (14% of diagnoses; by sub-type: 71 central and 2 others) and 34 cases of other specified and unspecified bone tumours (7%). The latter heterogeneous group of other specified and unspecified tumours was not analysed. Age standardised incidence rates per million population years for each of the bone cancer sub-groups were 2.97 (95% CI 2.59 - 3.35) for osteosarcoma; 0.78 (95% CI 0.60 - 0.96) for chondrosarcoma and 2.14 (95% CI 1.82 - 2.47) for Ewing sarcoma.

**Table 1:** Number of cases and age-standardised incidence rates (ASR) per million persons per year by diagnostic group, sex and age-group (in years), 1981-2002

			0 - 14		15 - 29		30 - 39	0 - 39
		N	ASR (95% CI)	N	ASR (95% CI)	N	ASR (95% CI)	ASR (95% CI)
**Osteosarcoma**	**All persons**	**82**	**2.48 (1.94,3.02)**	**135**	**4.53 (3.76,5.29)**	**19**	**0.98 (0.59,1.53)**	**2.97 (2.59,3.35)**
	**Male**	**45**	**2.66 (1.88,3.44)**	**83**	**5.56 (4.36,6.76)**	**14**	**1.44 (0.79,2.41)**	**3.51 (2.93,4.09)**
	**Female**	**37**	**2.30 (1.55,3.04)**	**52**	**3.49 (2.54,4.44)**	**5**	**0.52 (0.17,1.20)**	**2.42 (1.93,2.91)**
**Chondrosarcoma**	**All persons**	**2**	**0.06 (0.01,0.23)**	**36**	**1.17 (0.79,1.55)**	**35**	**1.80 (1.20,2.40)**	**0.78 (0.60,0.96)**
	**Male**	**1**	**0.06 (0.00,0.32)**	**23**	**1.50 (0.95,2.25)**	**23**	**2.38 (1.51,3.57)**	**1.00 (0.71,1.29)**
	**Female**	**1**	**0.07 (0.00,0.38)**	**13**	**0.83 (0.44,1.42)**	**12**	**1.22 (0.63,2.14)**	**0.55 (0.36,0.81)**
**Ewing sarcoma**	**All persons**	**73**	**2.24 (1.73,2.76)**	**85**	**2.85 (2.24,3.46)**	**8**	**0.41 (0.18,0.82)**	**2.14 (1.82,2.47)**
	**Male**	**40**	**2.42 (1.67,3.17)**	**54**	**3.62 (2.66,4.59)**	**4**	**0.42 (0.11,1.07)**	**2.51 (2.01,3.01)**
	**Female**	**33**	**2.06 (1.35,2.76)**	**31**	**2.08 (1.34,2.81)**	**4**	**0.41 (0.11,1.05)**	**1.77 (1.35,2.20)**

Incidence varied by age group (Table 1). Poisson regression models showed that after adjustment for sex and time period the incidence peaked among 15 - 29 year olds. The incidence rate ratios (IRRs) for those aged 15 - 29 years relative to 0 - 14 years were 2.04 (95% CI 1.55 - 2.68) for osteosarcoma; 22.33 (95% CI 5.38 - 92.73) for chondrosarcoma and 1.44 (95% CI 1.05 - 1.97) for Ewing sarcoma. The IRRs relative to the 0 - 14 age group for ages 30 - 39 were 0.45 (95% CI 0.28 - 0.75) for osteosarcoma; 34.34 (95% CI 8.26 - 142.76) for chondrosarcoma and 0.21 (95% CI 0.10 - 0.44) for Ewing sarcoma.

There were more male than female cases of all bone cancer combined (310 males, 199 females; *P *< 0.001) and for each of the sub-groups. The IRRs for males relative to females were 1.49 (95% CI 1.15 - 1.94) for osteosarcoma; 1.81 (95% CI 1.12 - 2.92) for chondrosarcoma and 1.42 (95% CI 1.04 - 1.93) for Ewing sarcoma.

The results of the analyses of time trends in bone cancer incidence during 1981 - 2002 are shown in Table 2. There was a statistically significant increase in the incidence of osteosarcoma of 2.54% per annum (95% CI 0.43 - 4.65; *P *= 0.02). There was little evidence for any temporal changes in the incidence rates for other diagnostic groups.

**Table 2:** Number of cases and age-standardised incidence rates (ASR) per million persons per year by diagnostic group, sex and time period

			1981 - 1988		1989 - 1995		1996 - 2002	%annual	
		N	ASR (95% CI)	N	ASR (95% CI)	N	ASR (95% CI)	change	**P**^**a**^
Osteosarcoma	All persons	82	2.65 (2.07,3.22)	71	2.85 (2.18,3.52)	83	3.51 (2.75,4.28)	2.54%	0.02
	Male	48	3.03 (2.17,3.88)	44	3.44 (2.41,4.46)	50	4.28 (3.08,5.47)	3.13%	0.08
	Female	34	2.27 (1.50,3.03)	27	2.26 (1.48,3.30)	33	2.74 (1.79,3.69)	1.75%	0.30
Chondrosarcoma	All persons	37	1.10 (0.75,1.46)	14	0.46 (0.25,0.78)	22	0.74 (0.46,1.13)	-0.80%	0.71
	Male	20	1.19 (0.72,1.83)	9	0.58 (0.26,1.11)	18	1.23 (0.72,1.97)	2.75%	0.51
	Female	17	1.02 (0.59,1.63)	5	0.35 (0.10,0.82)	4	0.26 (0.07,0.66)	-3.77%	0.14
Ewing sarcoma	All persons	73	2.44 (1.88,3.00)	42	1.75 (1.22,2.29)	51	2.21 (1.60,2.83)	-0.44%	0.72
	Male	42	2.77 (1.93,3.61)	29	2.40 (1.60,3.46)	27	2.37 (1.55,3.45)	-1.00%	0.47
	Female	31	2.11 (1.36,2.86)	13	1.09 (0.58,1.87)	24	2.06 (1.31,3.08)	0.51%	0.82

a. P value for test of no annual change

**Figure 1:**
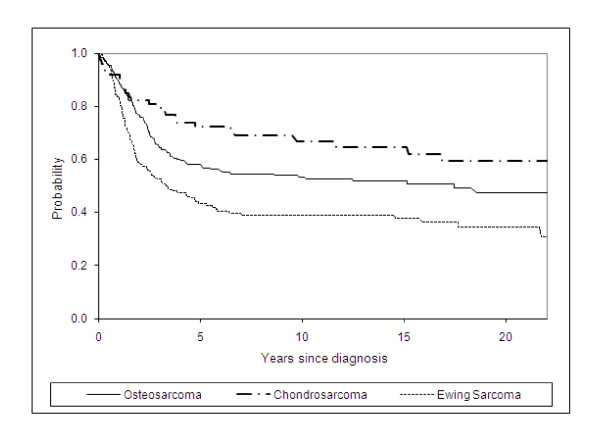
**Kaplan-Meier Survival by diagnosis group**.

Five-year survival rates by period of diagnosis by bone cancer sub-group are shown in Table 3. For all bone cancers combined 57% (95% CI 52 - 61) of 0 - 39 year olds survived for at least five years. Five-year survival rates for diagnostic sub-groups were 58% (95% CI 51 - 64) for osteosarcoma; 71% (95% CI: 59 - 81) for chondrosarcoma and 43% (95% CI 35 - 51) for Ewing sarcoma. There was evidence of significant differences in survival rates among the diagnostic groups (log-rank test: *P *< 0.001). Significantly better survival rates were found for chondrosarcoma compared with osteosarcoma (*P *= 0.001) and for osteosarcoma compared with Ewing sarcoma (*P *= 0.001) (Figure 1).

**Table 3:** Percentage five-year survival for 0-39 year olds by diagnostic group, sex and time period^a^

		1981 - 1987	1988 - 1994	1995 - 2000
		Rate (95% CI)	Rate (95% CI)	Rate (95% CI)
Osteosarcoma	All persons	49 (38,61)	61 (49,72)	62 (50,73)
	Male	47 (32,62)	54 (38,68)	53 (37,67)
	Female	54 (35,72)	71 (52,84)	76 (56,88)
Chondrosarcoma	All persons	71 (51,85)	67 (43,83)	76 (49,90)
	Male	75 (42,91)	63 (36,82)	77 (45,92)
	Female	69 (42,86)	80 (25,96)	75 (18,96)
Ewing sarcoma	All persons	37 (26,50)	46 (32,60)	49 (33,65)
	Male	35 (22,52)	47 (30,65)	50 (29,71)
	Female	40 (23,60)	44 (22,69)	47 (25,70)

a. Rates data include 95% CI in parentheses

**Figure 2:**
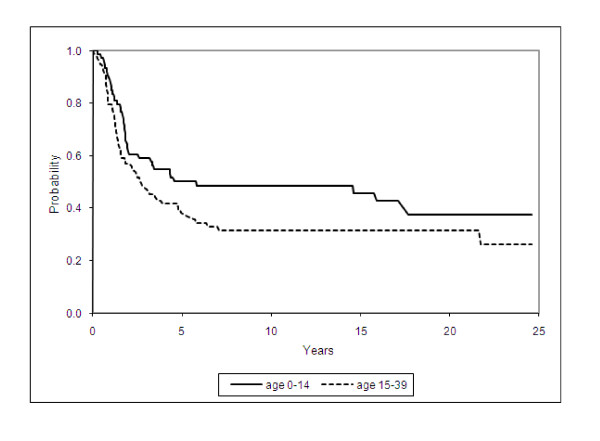
**Kaplan-Meier Survival for Ewing Sarcoma by age-group (in years)**.

Ewing sarcoma exhibited a higher risk of death for subjects aged 15 - 39 than those aged 0 - 14 (HR = 1.46 95% CI 0.98 - 2.17; *P *= 0.06) (Figure 2). After adjusting for age in the Cox model, the overall risk of death was estimated to decrease by 2.8% for each increasing year of diagnosis (HR per annum = 0.97 95% CI 0.94 - 1.00; *P *= 0.06). There was no evidence for differences in survival from Ewing sarcoma between males and females (*P *= 0.83).

For osteosarcoma there was a non-statistically significant improvement in survival during the study period (HR per annum = 0.98; 95% CI 0.95 - 1.01; *P *= 0.18). There was no significant evidence that sex (*P *= 0.11) or age (*P *= 0.50) had any effect on survival. Survival rates for chondrosarcoma remained constant (HR per annum = 1.00; 95% CI 0.94 - 1.07; *P *= 0.89) and there was no evidence that sex (*P *= 0.46) or age (*P *= 1.00) had any significant effect on survival.

## Discussion

This study of primary bone cancer in 0 - 39 years olds in northern England is the most recent analysis that has examined incidence and survival for this age group. Cases were limited to those aged less than forty years because this is the upper age-limit for entry into most clinical trials [16,23]. Furthermore, this age range coincides with the peak age-incidence distribution for the majority of malignant primary bone tumours. It is likely that a substantial proportion of the 0 - 39 year old patients have been treated according to trial protocols (although we did not have access to data on trial entry). The basic case incidence and survival data were accurately recorded by the registry. However, data on stage and treatment were not consistently and reliably recorded. Therefore, some of the differences in survival may be due to differences in case mix with respect to stage or treatment regime.

Although there is some evidence for an improvement in survival from Ewing sarcoma during the period 1981 - 2000, cases aged 15 - 39 years had worse outcome than those aged 0 - 14 years. In contrast, for osteosarcoma, there was no significant change in survival throughout the study period and no evidence that cases aged 15 - 39 years had worse outcome than childhood cases (aged 0 - 14 years).

The incidence rates reported in the present study were similar to those reported in other countries with mainly white populations. In addition, the predominance of osteosarcoma and Ewing sarcoma found in this study are also typical patterns reported in ethnically similar populations (5% were minority ethnic population) [24,25].

Incidence of osteosarcoma, chondrosarcoma and Ewing sarcoma was higher among males than females. This is consistent with previous literature, which has reported that although males and females tend to show similar incidences in childhood [11,15,24], when an extended age range is included, all three diagnostic groups are more common in males [25].

Osteosarcoma and Ewing sarcoma both had incidence peaks in the 15 - 29 age group. This is consistent with previous studies, which have widely reported incidence of these two diagnostic groups to rise after the onset of puberty, when young people are undergoing a growth spurt and bones experience rapid growth [8,11,24].

This study found an increase in the incidence of osteosarcoma, but no temporal changes in the incidence of Ewing sarcoma and chondrosarcoma during the period 1981 - 2002. In both the USA and Europe, overall bone cancer incidence across all age groups has been reported to have remained steady over the last 30 years [25,26].

We have previously analysed the survival of children resident in northern England and the West Midlands during the same time period [15]. For children we found that whilst there was an improvement in survival from Ewing sarcoma, there was no improvement for osteosarcoma. The present study confirms that the lack of improvement in survival for osteosarcoma also applies at ages 15 - 39 years. However, it has indicated a worse outcome for Ewing sarcoma cases in age group 15 - 39 years. Another recent study from the USA analysed data from the Surveillance, Epidemiology and End Results Program and has found that there has been no statistically significant improvement in survival from osteosarcoma (at all ages) from 1984 to 2004 [27].

The most recent national data from Great Britain reported five-year survival rates of 53% and 51% for cases aged 0 - 39 years during 1990 - 1994 [16]. Our data have shown that survival for northern England was better for osteosarcoma (58%) but worse for Ewing sarcoma (43%). Data for patients of all ages, from the United States National Cancer Database, also reported worse five-year survival (51.2%) for osteosarcoma and better five-year survival (50.2%) for Ewing sarcoma [28]. Better survival of osteosarcoma compared with the US study is likely to be due to the exclusion of patients aged more than 40 years from the present study. Another study of osteosarcoma from Finland reported five-year survival of 65% during 1981 - 1990 [29]. However, survival rates in northern England were similar to 15 - 24 year olds from a multi-centre (twenty countries) European study, which found five-year survival rates of 58% for osteosarcoma and 42% for Ewing sarcoma [30].

A number of studies have reported worse survival from osteosarcoma in adults aged more than forty years [27,28,31,32]. It is very well recognized that those aged more than 40 years have poorer prognosis than younger patients [33]. In our study of 0 - 39 year olds, we only found worse outcome for cases of Ewing sarcoma aged 15 - 39 years, but not for osteosarcoma. However, some other studies have specifically studied adolescent and young adult cases and have indicated worse outcome in this age group for both osteosarcoma and Ewing sarcoma compared with children [22,34,35].

The worse survival in cases of Ewing sarcoma aged 15 - 39 years is consistent with previous studies and may be due to a number of different factors including treatment, delays in diagnosis, metastatic disease, site and the stage of the tumour [36-39]. We were not able to investigate these factors within the scope of the present study. However, these issues will be addressed in future studies that focus on the Teenage and Young Adult (TYA) age group. A special National Cancer Research Institute TYA Clinical Studies Development Group has been formed and will oversee analyses of a new national TYA cancer dataset.

## Conclusion

This study has found an increase in the incidence of osteosarcoma, but no change in the incidence of Ewing sarcoma or chondrosarcoma. There was a lack of improved survival from osteosarcoma in both childhood and young adult cases and worse survival in Ewing sarcoma patients aged 15 - 39 years compared to those aged 0 - 14 years. Further research is needed to elicit the reasons for the failure to improve survival for osteosarcoma patients and for worse survival in older Ewing sarcoma patients.

## Competing interests

The authors declare that they have no competing interests.

## Authors' contributions

RE, RGF, PWJ, EM and RJQM contributed to the design of the study, the writing of the manuscript and the analysis and interpretation of data. KB, DF, PAM and MSP contributed to the writing of the manuscript and the interpretation of data. All authors read and approved the final manuscript.

## Pre-publication history

The pre-publication history for this paper can be accessed here:

http://www.biomedcentral.com/1471-2407/10/357/prepub
